# Contribution of Peripheral Injuries to the Symptom Experience of Patients with Mild Traumatic Brain Injury

**DOI:** 10.1089/neur.2021.0012

**Published:** 2021-08-06

**Authors:** Andrew M. Bryant, Michael A. McCrea, Lindsay D. Nelson

**Affiliations:** Departments of Neurology and Neurosurgery, Medical College of Wisconsin, Milwaukee, Wisconsin, USA.

**Keywords:** injury severity, mild traumatic brain injury, neurotrauma, peripheral injuries, polytrauma, outcomes

## Abstract

Peripheral injuries are common in patients who experience mild traumatic brain injury (mTBI). However, the additive or interactive effects of polytrauma on psychosocial adjustment, functional limitations, and clinical outcomes after head injury remain relatively unexamined. Using a recently developed structured injury symptom interview, we assessed the perception and relative importance of peripheral injuries at 3 months post-injury in patients with mTBI as defined by the American Congress of Rehabilitation Medicine. Our sample of Level 1 trauma patients (*n* = 74) included individuals who were treated and released from the emergency department (*n* = 43) and those admitted to an inpatient unit (*n* = 31). Across the sample, 91% of patients with mTBI experienced additional non-head injuries known to commonly impact recovery following mTBI, a majority of whom ranked pain as their worst peripheral injury symptom. Forty-nine percent of the mTBI sample (54% of the subsample with concurrent mTBI and peripheral injuries) reported being more bothered by peripheral injury symptoms than mTBI. Differences between patients with mTBI with worse mTBI symptoms versus those with worse peripheral injury symptoms are described. Conventional measures of injury severity do not capture patients' perceptions of the totality of their injuries, which limits the development of patient-centered treatments. Future research should enroll patients with mTBI diverse in peripheral injury severity and develop standardized assessments to characterize peripheral symptoms, enabling better characterization of the relevance of concurrent injuries in recovery and outcomes of patients with mTBI.

## Introduction

Traumatic brain injury (TBI) is a major cause of morbidity and mortality in the United States and worldwide.^1–3^ The overwhelming majority of TBIs are classified as mild TBI (mTBI).^1^ In addition to the functional impairments due directly to mTBI, approximately 70% of patients with mTBI who present to the hospital emergency department (ED) have additional non-TBI (i.e., peripheral) injuries.^4^ Despite the common co-occurrence, the contribution of peripheral injuries to the experiences and outcomes of patients with mTBI is poorly understood. Peripheral injuries have been understudied partly by design, as many prior mTBI studies excluded patients with significant polytrauma and/or compared mTBI patients with orthopedically injured (OI) control groups without mTBI, in an attempt to isolate the specific effects of brain injury.^5,6^

Although comparison of mTBI with OI groups is helpful for some research goals (e.g., identifying brain-specific clinical tests or blood-based biomarkers^7,8^), this practice does not allow one to characterize the patient experience, which comprises the totality of all injuries experienced. Further, such research practices rely on unrealistic assumptions that the effects of brain and peripheral injuries are additive and that peripheral injuries do not causally impact brain function. Such assumptions of mind-body dualism have been disproven in animal models (e.g., experimental peripheral nerve injury in animals results in inflammatory and neuroplastic markers within the brain^9^) and human studies (e.g., when comparing patients with mTBI to OI controls, both groups have increases in some plasma inflammatory cytokines^10^).

Peripheral injuries have also been ignored or eliminated as predictors in studies examining factors of mTBI recovery, with predictive variables such as clinical signs and symptoms of mTBI, and demographic and medical history variables predominating such investigations.^11,12^ A number of factors contribute to poor understanding of the potential additive or interactive effects of peripheral injuries on psychosocial adjustment, functional limitations, and clinical outcomes in patients with mTBI. As mentioned, exclusionary criteria based on peripheral injuries limits the generalizability of samples to the broader mTBI population, especially subpopulations of mTBI patients who present for higher levels of care. Additionally, some widely used outcome measurement techniques (e.g., versions of the Glasgow Outcome Scale) attempt to exclude the impact of peripheral injuries on outcomes. Finally, methods for characterizing peripheral injuries and their severity are limited and restricted to injury severity scores derived from chart review (i.e., Abbreviated Injury Scale [AIS], Injury Severity Score [ISS]), which were designed to predict mortality but correlate only weakly with patients' perceived injury severity or markers of injury morbidity.^13–15^

As a preliminary step toward advancing knowledge of the role of peripheral injuries in the recovery of patients with mTBI, we interviewed patients with mTBI at 3 months post-injury to assess the perception and relative importance of peripheral injuries. In characterizing the experiences and concerns of patients with mTBI about concurrent peripheral injuries, the findings might lead to improved awareness of the relevance of peripheral injuries in patient outcomes, could fuel additional research to improve the measurement of peripheral injuries, and may facilitate more patient-centered clinical care and clinical trial design.

## Methods/Results

Patients with mTBI (*n* = 74) were recruited from a Level 1 trauma center within 2 weeks of injury and were assessed at 3 months post-injury, as described in an earlier publication from this study.^16^ Inclusion criteria were 18 or more years of age, English-speaking, able to provide informed consent, and mTBI within 2 weeks of enrollment as defined by the American Congress of Rehabilitation Medicine.^17^ Because it was anticipated that clinical diagnoses of mTBI would often be missed, subjects were identified by a traumatic cause of injury and screened for eligibility using a semi-structured clinical interview to verify acute injury characteristics consistent with the definition of mTBI (e.g., unconsciousness, peri-traumatic amnesia, other signs of altered mental status). Medical chart review was also performed to verify objective signs commonly used to classify mTBI (e.g., Glasgow Coma Scale [GCS] score <15, acute intracranial findings found on head computed tomography [CT] scans).

This study was approved by the Medical College of Wisconsin's Institutional Review Board. At enrollment (<2 weeks post-injury), participants completed a demographic questionnaire and the first three items of the Rivermead Post Concussion Symptoms Questionnaire (RPQ-3; i.e., headaches, dizziness, nausea/vomiting) to estimate early TBI symptom severity.^18^ Responses on the RPQ-3 (possible range 0–12) were summed to yield a total score, after rescoring responses of 1 (“No more of problem” than pre-injury) to 0, as is customary for the instrument. Table 1 presents sample and injury characteristics. In summary, the sample was 57% male, mean [M] age = 45.2 years, (standard deviation [SD] = 15.4) and had an M estimated verbal intellectual functioning in the average range (education M = 12.9 years, SD = 2.0; Wide Range Achievement Test [WRAT]-4 Word Reading^19^ standard score M = 93.5, SD = 15.7). To enable study of patients with mTBI in different levels of care, recruitment roughly equally covered patients who were treated and released from the ED (58%) and those who were admitted to an inpatient unit (INP; 43%).

**Table 1.** Characteristics of Patients with Mild TBI (*n* = 74^a^) Reporting Worse TBI Symptoms (mTBI_Sx_) or Worse Peripheral Injury Symptoms (PER_Sx_)

	Sample		mTBI_Sx_ (*n* = 31)	PER_Sx_ (*n* = 36)	
Variable	M (SD) or* n *(%)	Range	M (SD) or* n *(%)	M (SD) or* n *(%)	P-value^b^
Age, years	45.19 (15.43)	18–86	43.48 (15.86)	47.28 (15.21)	0.322
Gender, male	42 (57%)	―	14 (45%)	26 (72%)	**0.024**
Race					0.854^c^
Black or African-American	32 (43%)	―	14 (45%)	14 (39%)	
White	37 (50%)	―	15 (48%)	20 (56%)	
Unknown/not reported	5 (7%)	―	2 (6%)	2 (6%)	
Education, years	12.92 (2.03)	8–18	13.13 (2.42)	12.72 (1.81)	0.445
WRAT Word Reading, standard score	93.49 (15.68)	37–135	94.23 (13.46)	93.39 (17.80)	0.831
History of headache, yes	20 (30%)	―	9 (29%)	11 (31%)	0.892
History of psychiatric disorder, yes	19 (28%)	―	10 (32%)	9 (25%)	0.511
Highest level of care					0.084^c^
Emergency department	43 (58%)	―	21 (68%)	16 (44%)	
Inpatient unit	31 (42%)	―	10 (32%)	20 (56%)	
Cause of injury					**0.031** ^c^
Motor vehicle/Traffic accident	47 (64%)	―	16 (52%)	27 (75%)	
Fall	16 (22%)	―	8 (26%)	7 (19%)	
Assault	6 (8%)	―	5 (16%)	0 (0%)	
Struck by/against	4 (5%)	―	2 (7%)	1 (3%)	
Other	1 (1%)	―	0 (0%)	1 (3%)	
GCS score					0.329^c^
15	38 (51%)	―	18 (58%)	17 (47%)	
<15	13 (18%)	―	4 (12%)	9 (25%)	
Missing	23 (31%)	―	9 (29%)	10 (28%)	
Head CT outcome					0.767^c^
Positive	18 (24%)	―	7 (26%)	10 (28%)	
Negative	31 (42%)	―	13 (42%)	15 (42%)	
No imaging	25 (34%)	―	11 (35%)	11 (31%)	
RPQ-3	2.36 (2.98)	0–11	3.48 (3.55)	1.61 (2.30)	**0.015**
Loss of consciousness, yes	43 (58%)	―	18 (58%)	21 (58%)	0.647
Post-traumatic amnesia, yes	45 (61%)	―	22 (71%)	19 (53%)	0.147
Injury Severity Score^d^	17.10 (9.81)	4–43	16.70 (9.80)	17.95 (9.83)	0.745
Abbreviated Injury Scale–Head & Neck score^d^	1.74 (1.81)	0–5	2.30 (2.16)	1.45 (1.64)	0.239
Abbreviated Injury Scale–Worst Peripheral score^d^	2.48 (1.15)	0–5	1.80 (1.23)	2.95 (0.76)	**0.004**
History of previous mTBI, yes	41 (55%)	―	16 (52%)	15 (42%)	0.468
What percent of your injury symptoms are TBI-related?	51.18 (26.58)	5–98	69.77 (12.92)	30.77 (18.75)	**0.000**
Current pain rating	2.55 (2.84)	0–10	2.32 (2.81)	2.97 (2.91)	0.358

Significant p-values (<.05) are in bold.

^a^ Seven patients did not endorse any additional non-TBI injuries and were excluded in between-group analyses.

^b^ *P*-values are from chi-square or independent samples *t* tests unless otherwise noted.

^c^ Fisher's exact test.

^d^ Injury Severity Score/Abbreviated Injury Scale was available for *n* = 30 (22 males and 8 females).

CT, computed tomography; GCS, Glasgow Coma Scale; M, mean; mTBI, mild traumatic brain injury; RPQ-3, sum of first three items of the Rivermead Post Concussion Symptom Questionnaire; SD, standard deviation; TBI, traumatic brain injury; WRAT, Wide Range Achievement Test.

To gain a better understanding of the experience of the patient with TBI, the Structured Interview of TBI Symptoms (SITS; see additional details in research by Emmert and colleagues^20^) was completed as part of a comprehensive in-person clinical outcome assessment at 3 months post-injury (for a complete list of assessments, see research by Harfmann and associates^16^). In brief, the SITS consists of several parts: 1) open-ended questions inquiring about symptoms experienced due to injury; 2) a structured, closed-ended interview of 31 symptoms (i.e., yes/no) worsened by mTBI (comprising the symptoms of several widely used mTBI symptom checklists); 3) rankings of the top three TBI and top three non-TBI symptoms; 4) comparisons between TBI and non-TBI symptoms; and 5) questions about the course and severity of symptoms.

**FIG. 1.**
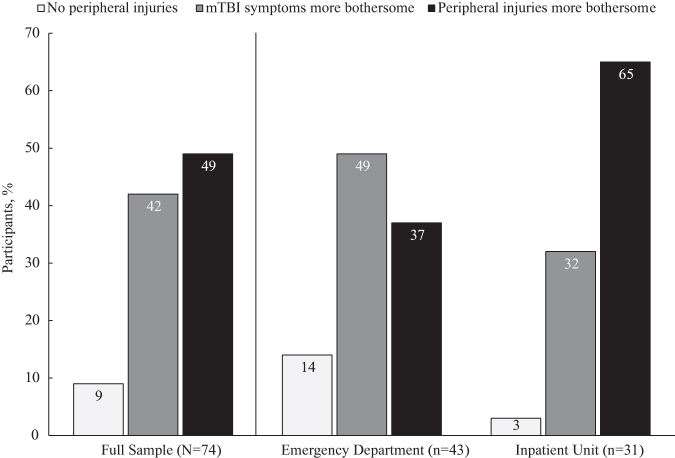
Percentage of participants who reported being more bothered by mTBI symptoms or peripheral injuries. mTBI, mild traumatic brain injury.

Of interest to the current study, the SITS posed two questions asking participants to compare their TBI and non-TBI injury symptoms: *“Have your other (non-TBI) injuries been more bothersome than your TBI symptoms?”* and *“What percentage of your injury symptoms are related to TBI (vs. other injuries)?”* Across our sample, 49% (*n* = 36) reported that peripheral injuries (i.e., non-TBI injury symptoms) had been more bothersome than mTBI symptoms (PER_Sx_), 42% (*n* = 31) reported that peripheral injuries had been less bothersome than mTBI symptoms (mTBI_Sx_), and 9% (*n* = 7) reported no injuries other than mTBI (see Fig. 1). The 7 participants with no peripheral injuries were eliminated from further analyses.

To explore possible group differences, Table 1 provides unadjusted statistical comparisons of the mTBI_Sx_ and PER_Sx_ groups on demographic and injury variables. *P*-values under 0.05 were considered significant. Consistent with expectations, individuals in the mTBI_Sx_ group, who reported that their mTBI symptoms were more bothersome than peripheral injury symptoms, also estimated that a significantly higher percentage of their injury symptoms were related to mTBI compared with those in the PER_Sx_ group (69.8% vs. 30.8%) although the sample as a whole varied widely in the percentage of injury symptoms attributed to mTBI (range 5–98%) even within group status (mTBI_Sx_ range 50–98%; PER_Sx_ range 5–80%). Interestingly, there were no differences between mTBI_Sx_ and PER_Sx_ groups on acute signs of TBI injury severity such as loss of consciousness (LOC), post-traumatic amnesia (PTA), or head CT findings. There was a trend for the PER_Sx_ group to be more likely to be admitted to the hospital (*p* = 0.084; see Fig. 1). Although only available from inpatient participants (*n* = 30), there were no differences on head AIS score between mTBI_Sx_ and PER_Sx_ groups, although the PER_Sx_ group had evidence of more severe peripheral injuries (i.e., higher maximum peripheral AIS score compared with the mTBI_Sx_ group, *p* = 0.004).

Within our study sample individuals more bothered by mTBI symptoms were more often female (*p* = 0.024). Follow-up analyses on other variables significantly different between mTBI_Sx_ and PER_Sx_ groups shown in Table 1 (i.e., RPQ-3, cause of injury, and percentage of injury symptoms attributed to mTBI) revealed that women reported more severe mTBI-related symptoms at enrollment (acute RPQ-3 = 3.67 vs. 1.68, *p* = 0.019) and attributed a higher percentage of injury symptoms related to mTBI (59.2% vs. 42.6%, *p* = 0.008), but did not significantly differ on cause of injury (*p* = 0.364) compared with men. Although highest peripheral AIS score was not significantly different between males and females (*p* = 0.351), it was only available for a subset of our sample (males, *n* = 22; females, *n* = 8), which precludes us from drawing general conclusions about the relationship between self-reported and objective peripheral injury severity across gender groups.

Across all participants who reported additional peripheral injuries, non-TBI injuries mostly occurred to the trunk region (79% of injuries were to the neck, shoulder, back, abdomen, hip, and/or thigh) followed by injuries to the upper extremities (e.g., arm, elbow, wrist, hand, fingers) in 34% of the sample and lower extremities (e.g., hip/thigh, knee, leg, foot, toes) in 45% of the sample. Those in the mTBI_Sx_ group had injuries to the trunk (68%), upper extremities (36%), and lower extremities (32%), and those in the PER_Sx_ group had injuries to the trunk (89%), upper extremities (33%), and lower extremities (56%). There were no differences in current general pain ratings at 3 months post-injury between the mTBI_Sx_ and PER_Sx_ groups.

To better understand the peripheral injury symptoms experienced by the entire sample, we coded interview responses at the 3-month follow-up visit to a question asking participants to report and rank their worst peripheral injury symptom. Based on a review of sample responses, the author team developed the following general categories to classify responses: bleeding/injury terminology, cosmetic concerns, loss of function, malaise/torpor/sleep, pain, sensory change, and unclassified. Although not all responses were characteristic symptom terms (e.g., loss of function, injury terms), we nevertheless coded all responses as is. Next, two raters independently coded each response into one of these categories, and a third was brought in to reconcile discrepant codes.

Of those with concurrent peripheral injuries, a significant majority (70%) designated pain (including general bodily pain and localized pain) as their top-ranked peripheral injury symptom. Loss of function (e.g., limited range of motion, stiffness, limitations in mobility) and bleeding/injury terminology (e.g., bleeding from cut, broken teeth) were ranked as the worst peripheral injury symptom types by 14% and 6% of our sample, respectively. Similar to the full sample, pain was the most prevalent primary peripheral injury symptom (mTBI_Sx_ = 68%; PER_Sx_ = 72%); however, stratifying by groups revealed that loss of function was the next most prevalent worst symptom for PER_Sx_ (22%), whereas bleeding/injury terminology and unclassified responses (e.g., “right arm”) were tied for the next most prevalent worst symptom for mTBI_Sx_ (9% each).

## Discussion

Our findings provide preliminary evidence that in Level 1 trauma patients with mTBI, peripheral injuries are more bothersome than mTBI symptoms in a substantial minority of patients treated and released from the ED (37%) and a majority of patients admitted to the hospital (67%). Across our sample, 91% of patients with mTBI experienced additional non-head injuries known to commonly impact recovery following mTBI.^21^ Many mTBI studies have attempted to isolate the effects of mTBI by excluding cases with significant peripheral injuries or by comparing TBI patients with other-injury controls leaving the additive or interactive effects of polytrauma on psychosocial adjustment, functional limitations, and clinical outcomes after injury relatively unexamined. The findings may serve as a reminder that methodology aimed at isolating the impact of TBI on clinical tests or outcomes substantially limits generalizability of findings to the broader mTBI patient population. These results, although preliminary from a modest sample at a single site, provide a more nuanced account of the role of peripheral injuries in the mTBI patient experience, a finding that we hope sparks more attention and measurement of such injuries in future TBI research.

There were no differences in acute signs of mTBI severity (i.e., GCS, LOC, PTA, CT findings) between those who were more bothered by their mTBI symptoms versus peripheral injuries. As one would expect, individuals with more severe acute mTBI symptoms (RPQ-3) within 2 weeks of injury were more likely to report being more bothered by mTBI symptoms at 3 months, whereas individuals with higher AIS-Peripheral scores were more likely to report being more bothered by peripheral symptoms. Interestingly, gender was a predictor of primary symptom concern (mTBI_Sx_ vs. PER_Sx_) due to women being more prevalent in the mTBI_Sx_ group. Women also reported significantly more acute/subacute mTBI-related symptoms (i.e., RPQ-3 score within 2 weeks of injury). These current findings may reflect the previously reported higher susceptibility of females to experience mTBI-related symptoms^22,23^ and supports prior findings that this phenomenon may be specific to mTBI symptoms rather than reporting bias or symptom-susceptibility more broadly.^24^

Conventional measures of injury severity (i.e., AIS and ISS), although invaluable, are limited in that they do not reflect the impact of peripheral injuries on the experience of patients with mTBI. Specifically, these measures are generally calculated for admitted patients only and were initially developed to determine risk of mortality.^13^ Consequently, the ISS is very weakly correlated with patients' perceived injury severity (*p* = 0.07) and symptoms.^14^ A recent study found that patients with TBI reported being more bothered by physical problems compared with OI controls despite no differences in peripheral ISS scores, further supporting that the ISS does not sufficiently capture patients' perceptions of injury.^16^ Patients have better outcomes when they are provided sufficient resources and information about the trajectory of their care.^6^ However, using ISS to estimate the impact of peripheral injuries may inadequately reflect specific patient concerns and limit a treatment team's ability to provide pertinent resources. Our brief self-report variable (i.e., which source of symptoms is more bothersome) correlated in expectable ways with head and peripheral AIS scores, which suggests that the self-report variable reflects injury severity, while more closely capturing patients' experience of the injury.

In summary, these preliminary data show that peripheral injuries are more bothersome to a large proportion of Level 1 trauma center patients with mTBI. Future research should develop and refine standardized assessments to characterize peripheral symptoms and better characterize how the totality of the injuries patients with TBI sustain affect outcomes such as functional outcomes and quality of life. Additionally, our findings on the general experience of participants in the first 3 months post-injury could be expanded to examine the trajectory of symptom recovery due to specific injuries. Doing so may lead to an improved understanding of the experience of patients with mTBI with concurrent peripheral injuries and refine patient-centered treatments (e.g., tailored rehabilitation vs. referrals to psychological services) for improved outcomes.

## Abbreviations Used

AIS: Abbreviated Injury Scale
CT: computed tomography
ED: emergency department
GCS: Glasgow Coma Scale
INP: inpatient unit
ISS: Injury Severity Score
LOS: loss of consciousness
M: mean
mTBI: mild traumatic brain injury
mTBI_Sx_: mTBI symptoms more bothersome
NIH: National Institutes of Health
OI: orthopedically injured
PCOR: Center for Patient Care and Outcomes Research
PER_Sx_: peripheral injury symptoms more bothersome
PTA: post-traumatic amnesia
RPQ-3: sum of first three items of the Rivermead Post Concussion Symptoms Questionnaire
SD: standard deviation
SITS: Structured Interview of TBI Symptoms
TBI: traumatic brain injury
WRAT: Wide Range Achievement Test
